# Delayed kidney response in AL amyloidosis: prevalence and clinical significance

**DOI:** 10.1093/ckj/sfag241

**Published:** 2026-08-05

**Authors:** Irene Martin Capon, Andrea Cifuentes, José E Ruiz-Cabello, Teresa Cavero, Pilar Auñón, Marina Alonso, Fernando Caravaca-Fontán, Eduardo Gutierrez, Manuel Praga, Enrique Morales, Ángel M Sevillano

**Affiliations:** Nephrology Department, Hospital Universitario 12 de Octubre, Madrid, Spain; Nephrology Department, Hospital Universitario 12 de Octubre, Madrid, Spain; Nephrology Department, Hospital Universitario 12 de Octubre, Madrid, Spain; Nephrology Department, Hospital Universitario 12 de Octubre, Madrid, Spain; Nephrology Department, Hospital Universitario 12 de Octubre, Madrid, Spain; Department of Pathology, Hospital Universitario 12 de Octubre, Madrid, Spain; Nephrology Department, Hospital Universitario 12 de Octubre, Madrid, Spain; Nephrology Department, Hospital Universitario 12 de Octubre, Madrid, Spain; Department of Medicine, Universidad Complutense, Madrid, Spain; Nephrology Department, Hospital Universitario 12 de Octubre, Madrid, Spain; Nephrology Department, Hospital Universitario 12 de Octubre, Madrid, Spain

**Keywords:** AL amyloidosis, anti-plasma cell therapy, chronic kidney disease, delayed response, kidney response

## Abstract

**Introduction:**

Kidney involvement is frequent in AL amyloidosis and significantly impacts prognosis. Although hematologic response is the recommended marker for monitoring treatment efficacy, organ-specific responses are frequently evaluated in clinical practice. In this context, the significance and optimal timing of kidney-specific response remain poorly defined in the literature.

**Methods:**

We conducted a retrospective study of patients with renal AL amyloidosis treated with or without anti-clonal therapy (ACT). Kidney response rates and their timing were analyzed. Patients were stratified by time to kidney response: very early (0–6 months), early (6–12 months), and late (>12 months). Baseline characteristics, treatments, and outcomes were compared across groups.

**Results:**

Seventy-six patients were included; 58 received ACT. Among them, 59% achieved hematologic response and 52% achieved kidney response. Kidney response was associated with improved kidney survival. Median time to hematologic response was 4.58 (1–9.38) months, whereas median time to partial and complete kidney response were 9 (6–18) and 16.5 (9–25) months (*P* = .03). Kidney outcomes were similar regardless of response timing. Patients with late kidney response had longer time to hematologic response (9.75 vs. 3 months, *P* = .02), longer treatment duration (24.5 vs. 9 months, *P* = .02), and received more ACT lines, particularly among those with very early hematologic response.

**Conclusion:**

Kidney response in AL amyloidosis occurs later than hematologic response but is associated with favorable kidney outcomes. In our study, delayed kidney response was associated with longer treatment duration despite hematologic response. Prospective studies are needed to determine whether treatment decisions based on kidney response alone improve or worsen patient outcomes.

KEY LEARNING POINTS **What was known:**

- Hematologic response is the primary therapeutic goal in AL amyloidosis, and its achievement is associated with better survival and organ outcomes.
- Kidney response is commonly used in clinical practice to monitor treatment response, but its prognostic value and optimal timing are not well established.
- The delay between hematologic and kidney response had been observed but not systematically analyzed in relation to treatment intensity or outcomes.

**This study adds:**

- Kidney response occurs significantly later than hematologic response in most patients with AL amyloidosis and renal involvement.
- Delayed kidney response is still associated with favorable kidney outcomes, including reduced need for kidney replacement therapy.
- The absence of kidney response, even after hematologic response, was associated with longer or more intensive therapy.

**Potential impact:**

- Lack of kidney response alone may not reflect ongoing disease activity and should be interpreted cautiously when guiding treatment decisions.
- These findings support the need for prospective studies to define the role of kidney response in treatment duration.

## INTRODUCTION

Amyloidosis encompasses a group of disorders characterized by the extracellular deposition of misfolded, insoluble proteins across various organs and tissues. Among the 18 recognized forms of systemic amyloidosis, immunoglobulin light chain (AL) amyloidosis is the most prevalent [1]. In AL amyloidosis, the precursor proteins are unstable immunoglobulin light chains produced by a clone of plasma cells or, less frequently, by a lymphoplasmacytic or mantle cell lymphoma. Although cardiac involvement is the primary prognostic determinant in AL amyloidosis, kidney involvement, that is present in over 50% of cases at diagnosis, also significantly influences the clinical evolution and outcomes of affected patients [2, 3].

Management of AL amyloidosis primarily aims to eradicate the underlying plasma cell dyscrasia, with the goal of halting amyloid fibril production and limiting progressive organ damage. Accordingly, anti-plasma cell therapy (ACT), comprising systemic chemotherapy with or without autologous hematopoietic stem cell transplantation (ASCT), represents the cornerstone of disease management [4]. Although ASCT is considered first-line therapy, only ∼20% of patients are eligible due to age or comorbidities [4–7].

Hematologic response in AL amyloidosis is determined by monitoring serum free light chains (FLC) and/or monoclonal protein levels. Among affected organs, cardiac involvement represents the most powerful determinant of prognosis [8, 9]. This recognition has led to the development of organ-specific assessment strategies. In this context, cardiac response is routinely evaluated using NT-proBNP and troponins [10], while kidney response evaluation includes serum creatinine (SCr), estimated glomerular filtration rate (eGFR) and proteinuria levels. Traditionally, kidney response has been defined by a ≥50% reduction in proteinuria without deterioration in kidney function [11]. More recent studies have proposed alternative definitions incorporating deeper reductions in proteinuria (>75% or >95%) and early eGFR changes as potential dynamic markers of renal response [8, 9, 12].

Clinical guidelines recommend an initial evaluation of the disease response 3–6 months after initiating treatment [11]. Nonetheless, the optimal timing for evaluating kidney response and its prognostic significance is not well established.

In this study, we describe the time to achievement of kidney and hematologic response in a cohort of patients with kidney AL amyloidosis, and discuss the implications of these findings in the management of the disease.

## MATERIALS AND METHODS

### Study population and design

All the patients in our center with a kidney-biopsy-proven AL amyloidosis between 1990 and 2024 were included in this study. Patients diagnosed with AA amyloidosis or other types of amyloidosis were excluded. All patients in this study had kidney involvement confirmed with a kidney biopsy with amyloid deposits on the kidney tissue. All study patients had an underlying monoclonal protein demonstrated by serum and/or urinary immunofixation. Patients were classified according to receipt of anti-plasma cell therapy and stratified by time to kidney and hematological response.

This study was conducted in accordance with the Declaration of Helsinki. Given the retrospective nature of the study, a waiver of informed consent from individual patients was granted.

### Clinical and laboratory data

Following a standardized protocol, data from included patients were extracted from medical records at disease onset and at the end of follow-up. Patients were followed according to routine clinical practice, with clinical and laboratory assessments performed approximately every 3–6 months. Recorded clinical variables included age, sex, kidney presentation, and extra-kidney manifestations. Laboratory parameters comprised SCr, eGFR (ml/min/1.73 m²), and 24-h urinary protein excretion. Treatments received during follow-up, especially chemotherapy, were also recorded. Time to hematologic response, time to kidney response, need for kidney replacement therapy (KRT), and time to death were also determined.

### Diagnosis of AL amyloidosis and definitions

The diagnosis of amyloidosis was established by the demonstration in light microscopy of Congo red deposits with apple-green birefringence on tissue biopsy samples. In all the cases, immunohistochemistry for AA amyloidosis was negative and immunofluorescence microscopy showed Ig light chain restriction. Electron microscopic features of randomly disposed, rigid, nonbranching, and variably long fibrils were also considered diagnostic.

Hematologic response was defined as a 50% reduction in measurable monoclonal protein levels (partial response), or complete eradication by immunofixation (complete response). Given the long study period, hematologic responses were retrospectively categorized based on the available laboratory data in medical records, using serum FLC–based criteria when these measurements were available. Partial kidney response was defined as a ≥50% reduction in initial proteinuria in the absence of a worsening of SCr or eGFR ≥25% from baseline. Complete kidney response was defined as proteinuria <0.5 g/24 h in the absence of worsening SCr or eGFR ≥25% from baseline [4, 12]. According to the time from treatment initiation to kidney response, patients were categorized as having very early kidney response (0–6 months), early kidney response (6–12 months), and late kidney response (>12 months). Also, the patients were stratified according to time to hematologic response in very early (0–6), early (6–12), and late (>12 months) hematological response groups. End stage kidney disease (ESKD) was defined as the presence of an eGFR under 15 ml/min/1.73 m^2^, the need for KRT or a pre-emptive kidney transplantation. Kidney baseline was defined as the time of AL amyloidosis diagnosis. The eGFR was calculated using the CKD-EPI formula. Acute kidney injury (AKI) at baseline was defined according to KDIGO guidelines criteria [13].

### Outcomes

The primary outcome was the time from treatment initiation to kidney response. Secondary outcomes included the proportion of patients achieving hematologic and kidney response, time to hematologic response, kidney and overall survival, and treatment duration.

### Statistical analysis

This was a retrospective, observational cohort study. Categorical data were expressed as absolute numbers and percentages. Continuous data were expressed as mean and standard deviation (SD) and, in the case of non-normal distribution, as median and interquartile range (IQR). The Kolmogorov-Smirnov test was used to test for normality. Comparisons were made using the *t test* for normally distributed continuous variables and the Mann-Whitney *U* test for non-normally distributed continuous variables. The chi-square or Fisher’s exact test were employed to compare the qualitative variables. The statistical significance level was set at *P* < .05. Kaplan-Meier survival analysis and the log-rank test were used to compare the cumulative incidence of dialysis among patients who received and those who did not receive treatment. For the analysis of continuous quantitative variables that follow a normal distribution in three groups, the ANOVA test was used, while for variables that did not follow a normal distribution, the Kruskal-Wallis test was applied. In cases where statistically significant differences were detected, a post hoc analysis using Dunn’s test and Bonferroni correction was performed to identify which groups were different. Cox proportional hazards regression models were used to analyze the main determinants of kidney survival. Covariables in multivariable models were selected on the basis of prior knowledge. Statistical analyses were conducted using IBM SPSS Version 22.0. A *P *< .05 was considered to be significant.

## RESULTS

### Baseline characteristics

Seventy-six patients with AL amyloidosis and kidney involvement were included in the study. Baseline characteristics are shown in Table 1. The main etiology of AL amyloidosis was monoclonal gammopathy of kidney significance IgG λ (MG IgGλ) (37%), followed by multiple myeloma IgGλ (MM IgGλ) (25%). Cardiac biomarkers were assessed in all patients and only seven had cardiac involvement expressed by an increase in their NT-proBNP.

**Table 1:** Baseline characteristics in patients with and without hematologic treatment.

		Patients included in the study	Patients receiving hematologic treatment	Patients not receiving hematologic treatment	*P*
*N*(%)		76 (100%)	58 (76%)	18 (24%)	
Types of AL amyloidosis *N* (%)	MG IgG *λ*	28 (37%)	17 (29%)	11 (61%)	.27
	MM IgG *λ*	19 (25%)	17 (29%)	2 (11%)	
	MG IgG *k*	9 (12%)	6 (10%)	3 (16%)	
	MM IgG k	5 (6%)	5 (9%)	0	
	MG IgM *k*	6 (8%)	5 (9%)	1 (6%)	
	MG IgA *λ*	1 (1%)	1 (2%)	0	
	MG IgA *k*	1 (1%)	1 (2%)	0	
	MM IgA *λ*	7 (10%)	6 (10%)	1 (6%)	
Age at the diagnosis of kidney amyloidosis, M ± SD (years)		65 ± 12	65 ± 11	64 ± 15	.12
Male/woman, *N* (%)		40/36 (53/47%)	34/24 (59/41%)	6/12 (33/67%)	.06
HTA, *N* (%)		12 (16%)	9 (15%)	3 (17%)	.91
DM, *N* (%)		11 (14%)	6 (10%)	5 (28%)	.07
Biochemical characteristics					
SCr, mg/dl		1.09 (0.87–1.77)	1.02 (0.81–1.72)	1.29 (0.97–2.26)	.07
eGFR, ml/min/1.73 m^2^		76.36 (40.25–96.21)	71.36 (40.25–98.75)	56.34 (31.33–80.76)	.05
Serum albumin, g/dl		2.9 (2.2–3.6)	2.91 (2.17–3.8)	3.01 (2.17–3.01)	.37
Proteinuria, g/day		5.01 (3.30–8.02)	4.81 (2.96–6.7)	7.05 (3.90–10.25)	.07
Kidney clinical manifestations at baseline					
AKI, *N* (%)		31 (41%)	21 (36%)	10 (56%)	.14
Nephrotic syndrome, *N* (%)		44 (58%)	33 (57%)	11 (61%)	.75
Histological characteristics at baseline					
Glomerular amyloid deposits		76 (100%)	58 (100%)	18 (100%)	–
Vascular amyloid deposits		30 (40%)	19 (33%)	11 (61%)	0.03
Glomerular sclerosis,					
- Number patients *N* (%)		32 (45%)	21 (36%)	6 (33%)	0.61
- Mean number of sclerosed glomeruli		10 (0–20)	10 (3.77–17.5)	10 (0–20)	
Interstitial fibrosis		23 (31%)	16 (27%)	7 (39%)	
- Mild		14 (18%)	11 (19%)	3 (17%)	
- Moderate		6 (8%)	3 (5%)	3 (17%)	0.45
- Severe		3 (5%)	2 (3%)	1 (5%)	

Data is presented as median (IQR) unless otherwise specified.

Abbreviations: DM, diabetes mellitus; HTA, hypertension; k: kappa, λ: lambda.

The mean age at diagnosis of kidney amyloidosis was similar between patients who received hematologic treatment (65 ± 11 years) and those untreated (64 ± 15 years), *P* = .12. The mean age at the diagnosis of the hematologic disease was 65 ± 11 years for patients under ACT and 64 ± 14 years for cases without it (*P* = .19). Therefore, 70% of patients were diagnosed with renal amyloidosis and the hematologic disorder simultaneously, while in 30% the diagnosis of renal involvement was delayed. The median time to the diagnosis of renal amyloidosis in these patients was 248 days (117–1018).

Patients who received anti-plasma cell therapy exhibited better kidney function, with a difference that approached statistical significance. Median SCr in patients with and without ACT was 1.02 (0.81–1.72) mg/dl and 1.29 (0.97–2.26) mg/dl, (*P* = .07). Median eGFR was 71.36 (40.25–98.75) ml/min/1.73 m^2^ and 56.34 (31.33–80.76) ml/min/1.73 m ^2^, *P* = .05.

The incidence of AKI at baseline was 36% and 56% (*P* = .14) in patients treated with and without hematologic therapy, respectively. However, the most frequent kidney involvement in these patients was nephrotic syndrome, which was observed in 44 (58%) of the included patients, without differences between treated and untreated populations (57% vs. 61%, *P* = .75).

Regarding histologic findings, the prevalence of glomerular and interstitial fibrosis was similar in both groups, with a median of 10 sclerosed glomeruli (*P* = .61) and a percentage of fibrosis of 27% and 39% in treated and untreated patients (*P* = .45). The presence of vascular amyloid deposits was significantly higher in patients who did not receive ACT (33% vs. 61% respectively, *P* = .03).

### Treatment

Fifty-eight (76%) of the included patients underwent hematologic-targeted treatment. The most common therapeutic strategy used was ASCT (26%), although a substantial proportion of patients received dexamethasone- and bortezomib-based combination regimens (DexBor and related protocols). The type of treatment received by each patient is displayed in Supplementary Table S1. Of the remaining 18 patients, 15 cases (83%) were not eligible for ACT due to advanced age, significant comorbidities, frailty, or poor functional status at diagnosis, as determined by the treating physician. In some cases, advanced disease stage and impaired kidney function also contributed to the decision not to initiate therapy. Three patients (17%) died before the initiation of hematologic therapy.

### Outcomes

**Figure 1:**
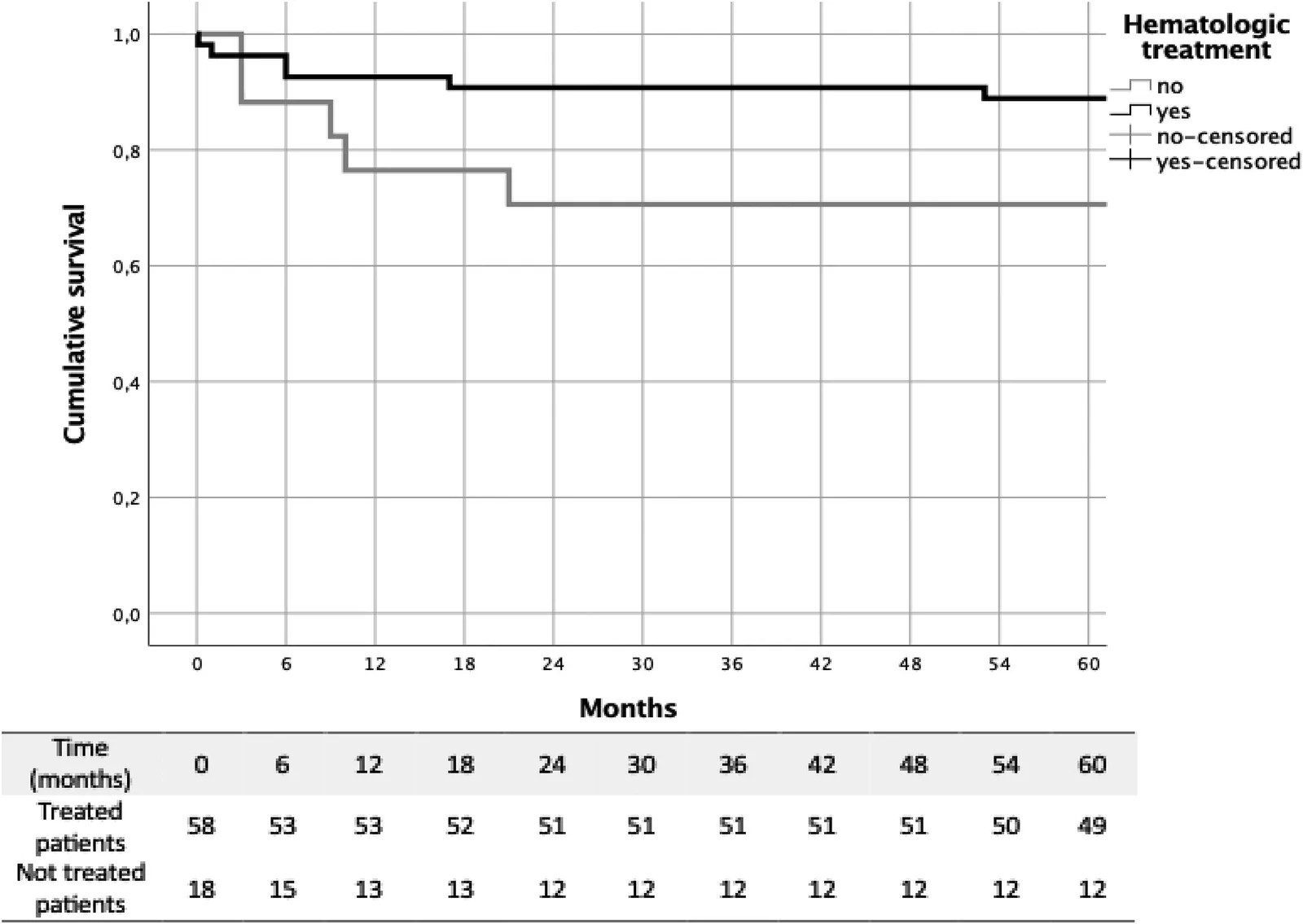
Kidney survival rate among patients receiving hematologic treatment and patients not receiving hematologic treatment. *(Log-rank test: χ² = 2.31, P = .01)*.

Thirty-four (59%) patients who received treatment in our cohort achieved hematologic response, and 30 (52%) achieved kidney response. The median time to hematologic response was 4.58 (1–9.38) months, while the median time to partial kidney response was 9 (6–18) months. Time to complete kidney response was 16.5 (9–25) months. None of the patients without ACT achieved hematologic or kidney response, showing at last follow-up a significantly lower eGFR and a higher proteinuria, compared to patients under hematologic treatment. This resulted in a higher need for KRT in untreated patients (50%) compared to treated patients (7%) (*P* = .01), Table 2. Time to ESKD was significantly longer in the treated group (*P* = .01), as is reflected in Figure 1. However, after adjustment for baseline kidney function, proteinuria, and AKI, treatment status was no longer significantly associated with kidney survival (Supplementary Table S2). In a sensitivity analysis additionally including hypertension and diabetes mellitus, the results remained unchanged, with baseline eGFR being the only variable independently associated with kidney survival (Supplementary Table S3). Likewise, in an exploratory multivariable model including baseline histological variables, none of the histological parameters evaluated were independently associated with kidney survival (Supplementary Table S4).

**Table 2:** Outcomes in patients with and without hematologic treatment.

	Patients included in the study	Patients receiving hematologic treatment	Patients not receiving hematologic treatment	*P*
*N* (%)	76 (100%)	58 (76%)	18 (24%)	–
Time of follow up, months	60 (9–131)	64.5 (9–120)	32.5 (9.75–312)	.70
SCr, mg/dl	1.90 (0.98 -4.22)	1.38 (0.90–2.77)	2.5 (2.5–5.7)	.01
eGFR, ml/min/1.73 m^2^	41.83 (18.31–88.25)	44.01 (44.01–89.24)	22.86 (8.30–39.78)	.02
Proteinuria, g/day	1.5 (0.22–5.01)	1.12 (0.19–4.35)	3.45 (1.39–9.48)	.02
Serum albumin, mg/dl	3.60 (2.70–4.10)	3.90 (2.85–4.12)	3.07 (2.50–3.55)	.08
Hematologic response, *N* (%)	34 (45%)	34 (59%)	0	.01
Kidney response, *N* (%)	30 (40%)	30 (52%)	0	.01
Time to hematologic response, months	4.58 (1–9.38)	4.58 (1–9.38)	No response	.01
Time to partial kidney response	9 (6–18)	9 (6–18)	No response	.01
Time to complete kidney response	16.5 (9–25)	16.5 (9–25)	No response	.01
ESKD, *N* (%)	17 (22%)	4 (7%)	9 (50%)	.05
Time to ESKD months,	21 (4.5–138)	58.5 (4.75–171)	10 (3–94)	.01
Death, *N* (%)	30 (40%)	27 (46%)	14 (78%)	.02
Time to death, months	13 (4–44)		25 (7.5–62.75)	

Data is presented as median (IQR) unless otherwise specified.

As shown in Supplementary Table S5, when the year of disease diagnosis was stratified by decade, the rate of kidney response increased significantly from 16% in 1990–2000 to 60% in 2010–24 (*P* = .02). This increase paralleled a marked rise in the proportion of patients receiving ACT over time (42% in 1990–2000 vs. 95% in 2010–24, *P* = .01), suggesting that advances in therapeutic strategies may have contributed to improved renal outcomes. In contrast, the time to achieve kidney response did not differ significantly between groups (*P* = .85), a finding that was further confirmed in sensitivity analyses. Specifically, in a Cox regression model adjusted for era of diagnosis, era was not independently associated with time to kidney response (HR 0.97, *P* = .95).

#### Patients with and without kidney response

Table 3 shows the baseline characteristics of patients with and without kidney response. The prevalence of ASCT was 43% in the group of patients with kidney response and 7% in patients without response (*P* = .01). There were no other baseline differences between both groups. Median follow-up was significantly longer in patients who achieved kidney response compared with those without response [91.5 (60–123) vs. 16.5 (3.5–105) months, *P* = .01], mainly reflecting the higher mortality observed in the non-response group. Table 4 displays the outcomes between patients who achieve and not kidney response. Patients who achieved kidney response demonstrated superior kidney function at the end of follow-up: better eGFR, 76 (32.06–95.45) vs. 34.27 (7.29–59.8) (*P* = .03), lower levels of creatinine, 1.22 (0.80–2.66) vs. 2.50 (1.25–5.70) (*P* = .04), and lower levels of proteinuria, 0.38 (0.14–1.5) vs. 3.50 (0.85–6.03) (*P* = .01). The need for KRT was significantly lower in patients who achieved kidney response (0% vs. 15%) (*P* = .03), as was mortality (28% vs. 69%) (0.01). A multivariate analysis was performed to assess whether kidney response was independently associated with kidney survival, Supplementary Table S6. The variables analyzed included achievement of hematologic response, achievement of kidney response, eGFR at diagnosis, baseline proteinuria, and age at disease diagnosis. Both kidney response (HR 0.15, *P* = .02), and higher eGFR at diagnosis (HR 0.96, *P* < .05) were independently associated with improved kidney survival.

**Table 3:** Baseline characteristics in treated patients according to kidney response status.

	Treated patients	Patients with kidney response	Patients without kidney response	*P*
*N (%)*	58	30 (52)	28 (48)	–
Age, M ± SD (years)	65.69 ± 11.67	63.71 ± 11.96	64.20 ± 11.37	.33
Male/woman, *N* (%)	34/24 (59/41)	15/15 (50/50)	19/9 (68/32)	.17
HTA, *N* (%)	9 (15)	4 (13)	5 (18)	.63
DM, *N* (%)	6 (10)	2 (7)	4 (14)	.45
Biochemical characteristics				
SCr, mg/dl	1.02 (0.81–1.72)	0.96 (0.72–1.42)	1.16 (0.80–1.95)	.35
eGFR, ml/min/1.73 m^2^	71.36 (40.25–98.75)	89.50 (51.92–100.57)	60.73 (38.19–97.35)	.15
Serum albumin, g/dl	2.91 (2.17–3.8)	3.30 (2.25–3.45)	2.70 (2.12–3.45)	.06
Proteinuria, g/day	4.81 (2.96–6.7)	4.81 (2.99–7.02)	4.82 (1.62–6.55)	.61
Kidney clinical manifestations at baseline				
AKI, *N* (%)	21 (36)	8 (27)	13 (46)	.12
Nephrotic syndrome, *N* (%)	33 (57)	17 (57)	16 (57)	.19
Histological characteristics at baseline				
Glomerular amyloid deposits	58 (100)	30 (100)	26 (100)	–
Vascular amyloid deposits	19 (33)	11 (36)	8 (29)	.51
Glomerular sclerosis,				.14
- Number patients *N* (%)	21 (36)	13 (43)	8 (29)	
- Mean number of sclerosed glomeruli	10 (3.77–17.5)	9 (0–15)	10.5 (9.25–29.5)	
Interstitial fibrosis	16 (27)	11 (36)	5 (18)	
-Mild	11 (19)	8 (27)	3 (11)	.19
-Moderate	3 (5)	1 (3)	2 (7)	
-Severe	2 (3)	2 (6)	0	

Data is presented as median (IQR) unless otherwise specified.

Abbreviations: DM, diabetes mellitus; HTA, hypertension.

**Table 4:** Outcomes in treated patients according to kidney response status.

	Patients with kidney response	Patients without kidney response	*P*
*N (%)*	30 (52)	28 (48)	–
SCr at last follow-up, mg/dl	1.22 (0.80–2.66)	2.50 (1.25–5.70)	.04
eGFR at last follow-up, ml/min/1.73 m^2^	76.00 (32.06–95.45)	34.27 (7.29–59.8)	.03
Proteinuria, g/day	0.38 (0.14–1.5)	3.50 (0.85–6.03)	.01
Serum albumin, mg/dl	3.62 ± 0.79	3.28 ± 1.11	.22
End stage kidney disease, *N* (%)	0	4 (15)	.03
Death rate, *N* (%)	8 (26)	18 (64)	.01
Time of follow up, months	91.5 (60–123.02)	16.5 (3.51–105.01)	.01

Data is presented as median (IQR) unless otherwise specified.

#### Patients classified according to time to kidney response

The baseline characteristics and outcomes of the patients with very early (0–6 months), early (6–12 months), and late (>12 months) kidney response are shown in Table 5. There were no differences in laboratory or histological baseline characteristics among the three groups. In exploratory analyses, baseline histological lesions were not associated with time to kidney response, including glomerulosclerosis (Spearman ρ = −0.21, *P* = .38), interstitial fibrosis (Kruskal-Wallis test, *P* = .16), and vascular amyloid deposits (Mann-Whitney test, *P* = .79). Time to hematological response was longer in the late kidney response group [9.75 (3.87–13.5) months] compared with early response group [3 (1–6.10) months], *P* = .02. None of the patients who achieved kidney response progressed to ESKD and kidney function and proteinuria at the end of follow-up were similar in the three groups. Regarding treatment, the proportion of patients who received two or three lines of ACT was higher in the late kidney response group, although the difference was non-significant. However, when the three groups were stratified by time to hematological response and the number of hematologic treatment lines was analyzed, it was shown that patients with very early hematological response (0–6 months) and late kidney response received significantly more lines of ACT than those with very early hematological response and very early or early kidney response groups, Supplementary Table S7. No other differences were found. The duration of ACT was also longer in the late kidney response group [24.5 (10.9–43.57) months] than in the early response group [9 (6–15.7) months], *P* = .02.

**Table 5:** Baseline characteristics and outcomes at last follow-up between patients with very early, early kidney, and late kidney response.

	Very early response 0–6 months	Early response 6–12 months	Late response >12 months	*P*
Baseline characteristics				
*N* (%)	7 (23)	13 (43)	10 (34)	
Age at the diagnosis of kidney amyloidosis, M ± SD (years)	71.14 ± 10.18	63.62 ± 11.35	60.1 ± 12.74	.17
SCr, mg/dl	1.05 (0.7–1.5)	0.94 (0.61–1.61)	0.95 (0.79–1.59)	.82
eGFR, ml/min/1.73 m^2^	80 (44.27–96.45)	89 (40.24–191.21)	90 (83.78–101.90)	.69
Serum albumin, g/dl	3.5 (3–3.9)	3.6 (2.25–4.15)	2.9 (2.07–4.02)	.74
Proteinuria, g/day	6.55 (3.30–8.9)	4.4 (2.2–6.15)	5.05 (2.8–7.89)	.29
AKI, *N* (%)	2 (28)	5 (38.5)	1 (10)	.31
Nephrotic syndrome, *N* (%)	3 (43)	8 (61)	6 (60)	.70
Glomerular amyloid deposits	7 (100)	13 (100)	10 (100)	–
Vascular amyloid deposits	3 (43)	4 (31)	4 (40)	.83
Glomerular sclerosis,				
- Number patients *N* (%)	4 (31)	7 (54)	2 (20)	.38
- Mean number of sclerosed glomeruli	12 (6–12)	9 (5–12)	5 (0–42)	.59
Interstitial fibrosis				
- Mild	3 (43)	4 (31)	1 (10)	.35
- Moderate	1 (14)	0	0	
- Severe	0	1 (8)	1 (10)	
Treatment				
Number of hematologic treatment lines, *N* (%)				
- 1 line of treatment	4 (57)	9 (69)	0	.23
- 2 lines of treatment	3 (43)	3 (23)	6 (60)	
- 3 lines of treatment	0	1 (8)	4 (40)	
Treatment time, months	9 (1.5–24)	9 (6–15.7)	24.5 (10.9–43.57)^a^	.07
Time to hematologic response, months	4 (1.38–18.65)	3 (1–6.10)	9.75 (3.87–13.5)^a^	.07
*N* patients with hematologic treatment after hematologic response	4 (57)	7 (53)	7 (70)	.60
Kidney outcomes				
End stage kidney disease, *N* (%)	0(0%)	0(0%)	0(0%)	.9
SCr, mg/dl	1.29 (0.68–3.42)	1.02 (0.81–2.99)	1.39 (0.83–2.45)	.87
eGFR, ml/min/1.73 m^2^	81 (20.31–98.03)	77 (43.81–92.39)	44.61 (31.55–90.11)	.65
Proteinuria, g/day	0.38 (0.17–1.80)	0.48 (0.12–1.43)	0.35 (0.18–2.92)	.86
Serum albumin, mg/dl (M ± SD)	3.9 (3.25–4)	3.85 (2.22–4.02)	4.2 (3.92–4.35)	.57

a Statistically significant differences between the kidney response groups of 6–12 months and >12 months.

Data is presented as median (IQR) unless otherwise specified.

## DISCUSSION

Advancements in the treatment of the hematological disorders underlying the amyloid production have dramatically improved outcomes in AL amyloidosis, leading to an increase in the survival of affected patients. Current guidelines recommend monitoring hematological parameters to assess treatment response in this disease [11]. However, kidney and cardiac involvement are critical determinants of the prognosis of AL amyloidosis and may not always align with hematological response. Therefore, in clinical practice, assessment of organ-specific response to ACT is routinely performed [9, 14]. While the role of troponins in assessing cardiac response is well established, the criteria for kidney response and its prognostic value remain less clearly defined.

In our cohort of patients with AL amyloidosis and kidney involvement, we have reported that traditional criteria for kidney response are associated with an improvement in kidney survival. Notably, this kidney response consistently occurred after hematologic response, which was observed exclusively in those patients under ACT. Indeed, untreated patients experienced an accelerated kidney function impairment and higher rates of ESKD (50%) compared to treated patients (7% and 46%, respectively). However, these findings should be interpreted with caution. Untreated patients in our cohort had worse baseline kidney function and a higher burden of comorbidities at diagnosis, and in multivariable models, treatment status was no longer independently associated with kidney survival. Additional sensitivity analyses including baseline comorbidities yielded similar results, and exploratory histological models did not identify independent associations between baseline kidney lesions and kidney survival. These findings suggest that baseline kidney severity, rather than treatment exposure alone, may explain the differences observed between treated and untreated patients.

Nevertheless, once hematologic control is achieved, previous studies and our own cohort suggest that deeper hematologic responses are closely linked to subsequent renal improvement. In that way, Leung et al. reported that, among a group of patients with AL amyloidosis and kidney involvement treated with ASCT, those who achieved a complete hematologic response showed a reduction in proteinuria >75% in 73.7% of cases and >95% in 52.6%. In contrast, only 25% of patients with partial hematologic response achieved >75% reduction in proteinuria, and none with less than very good partial response attained >95% reduction. Remarkably, our cohort showed a significant improvement in kidney response rates over time, reaching 60% response in the most recent decade (2010–24) compared to only 16% in the 1990s. It is noteworthy that patients who achieved kidney response had a significantly higher prevalence of ASCT (43%) compared to those without response (7%). These findings likely reflect improvements in therapeutic strategies, earlier diagnosis, and more accurate selection of candidates for intensive therapies.

However, patients in our study showed a significant delay between hematological and kidney response. In this way, whereas hematological response occurred relatively quickly after treatment initiation (median of 4.58 months), complete kidney response was observed at a median follow-up of 16.5 months (*P* = .03). Similar delays in the kidney response after ACT have been previously reported [8, 15–17].

The lag in kidney response relative to hematological response likely reflects the slow clearance of amyloid deposits from kidney tissue and subsequent functional recovery, often manifested as persistent proteinuria with stable kidney function. Indeed, the persistence of kidney amyloid deposits after hematological response has been well documented in repeated biopsy studies [17–20]. In addition, chronic structural lesions, such as glomerular sclerosis and interstitial fibrosis, in patients experiencing a delayed renal response, could contribute to persistent proteinuria, even after the resolution of amyloid deposits [21]. In this context, in patients with complete hematologic response without partial kidney response after one year of treatment, repeat kidney biopsy may provide additional information by identifying chronic damage or persistent amyloid deposits. Although these findings may be useful to predict kidney outcomes, their role in guiding treatment decisions despite hematological response remains uncertain [21].

In this study we stratified our patients according to the time to achieve kidney response (0–6 months, 6–12 months, and >12 months). We found no significant differences in their baseline characteristics that could predict a shorter or longer time to response. However, the patients with a late kidney response presented a more delayed hematological response compared to those with very early kidney response (3 months vs. 9 months). Remarkably, contrary to previous reports, in which an early response was associated with a better prognosis, the kidney outcomes were similar in the three groups [13].

Notably, patients with a delayed kidney response underwent more lines of ACT, which extended the time to hematological treatment by up to 24 months, despite the hematological response being achieved after a median of 9 months. Furthermore, among patients who achieved very early hematological response, those with late kidney response received significantly more ACT lines than those with very early and early kidney response. Although current guidelines do not recommend the use of organ-specific biomarkers beyond hematological response to guide ACT, our findings suggest that, in the clinical practice, the absence of kidney response despite hematologic control was associated with longer or more intensive hematological treatment administered to patients with primary amyloidosis. Although our retrospective data support association rather than causality, this treatment pattern may reflect attempts to improve kidney outcomes in patients without renal remission [21]. Nevertheless, as discussed above, most of these patients probably present with either established or chronic kidney damage or “old” AL amyloid deposits that have not yet been removed. Therefore, kidney response should be interpreted cautiously when used to guide treatment duration or intensity in clinical practice. However, prospective studies evaluating toxicity and relapse data are needed to clarify whether prolonged therapy is beneficial or potentially unnecessary.

The main limitations of our study include its retrospective nature, leading to heterogeneous treatment approaches, the lack of repeated kidney re-biopsies, and the long study period, during which response criteria evolved. In addition, the relatively small sample size, particularly within subgroup analyses according to time to renal response, may have limited the statistical power of our comparisons and these findings should therefore be interpreted cautiously. Accordingly, subgroup analyses based on timing of kidney response should be considered exploratory because of the small sample sizes However, the strengths of this study lie in the substantial patient cohort (76 individuals) and the extended, close clinical follow-up.

In summary, treatment advances in AL amyloidosis have improved kidney outcomes in patients with kidney involvement. The time to kidney response is significantly longer than the time to hematologic response, reflecting the slow clearance of amyloid deposits from kidney tissue. Nonetheless, kidney response is associated with good kidney outcomes, regardless of the time to achieve it. The absence of kidney response after hematological response may indicate a delayed kidney response or the presence of irreversible chronic damage. In our cohort, the delay in kidney remission was associated with prolonged/more intensive treatment. However, kidney response alone should be interpreted cautiously when guiding treatment decisions, as the absence of response may simply reflect delayed organ recovery rather than ongoing disease activity. Further prospective studies are needed to determine the optimal role of kidney response in guiding therapy duration.

## Supplementary Material

sfag241_Supplemental_File

## Data Availability

The data underlying this article are available in the article and in its online supplementary material.
