# Supplementary material for: Delayed kidney response in AL amyloidosis: prevalence and clinical significance
Source: Clin Kidney J. 2026 Aug 5;19(8):sfag241. doi: 10.1093/ckj/sfag241 (PMC13498870; doi:10.1093/ckj/sfag241)
Supplement: Supplementary file 1 [file sfag241_supplemental_file.docx]

**Supplementary material**

**Supplementary Table 1. Type of treatment received**

| Type of treatment | Number of patients (58) |
| --- | --- |
| ASCT | 15 (26%) |
| CyBorD | 5 (9%) |
| CyBor | 4 (7%) |
| BMDex | 6 (10%) |
| MDex | 9 (15%) |
| DexBor | 10 (17%) |
| DexBorLen | 3 (5%) |
| DexLen | 4 (7%) |
| CLenD | 2 (4%) |

*Abbreviations: ASCT (autologous hematopoietic stem cell transplantation), BMDex (bortezomib, melphalan, and dexamethasone), CLenD (cyclophosphamide, lenalidomide, dexamethasone),*

*CyBorD (cyclophosphamide, bortezomib, dexamethasone + Daratumumab), DexBor (dexamethasone, bortezomib), DexBorLen (dexamethasone, bortezomib, lenalidomide), DexLen (dexamethasone, lenalidomide), MDex (melphalan, and dexamethasone).*

**Supplementary Table 2. Multivariable Cox regression analysis evaluating the association between treatment and kidney survival**

| Variable | HR (CI 95%) | P |
| --- | --- | --- |
| Treatment | 0.71 (0.23 – 2.19) | 0.52 |
| eGFR at diagnosis | 0.97 (0.95-0.99) | 0.01 |
| Baseline proteinuria | 1.02 (0.98 – 1.06) | 0.26 |
| AKI at diagnosis | 1.10 (0.28 – 4.30) | 0.89 |

*Multivariable Cox regression model including treatment status, baseline kidney function, proteinuria, and AKI at diagnosis. P 0.02, the multivariate model is globally significant. Higher baseline eGFR was independently associated with a lower risk of progression to ESKD. Treatment status, baseline proteinuria, and AKI were not significantly associated with kidney survival after adjustment. HR are shown with their corresponding 95% CI and p-values. A HR < 1 indicates a lower risk of progression.*

*Abbreviations: HR, hazard ratio; CI, confidence interval; eGFR, estimated glomerular filtration rate; AKI, acute kidney injury; ESKD, end-stage kidney disease.*

**Supplementary Table 3. Sensitivity multivariable Cox regression analysis evaluating the association between treatment and kidney survival, including baseline comorbidities**

| Variable | HR (CI 95%) | P |
| --- | --- | --- |
| Treatment | 0.72 (0.24 – 2.18) | 0.55 |
| eGFR at diagnosis | 0.97 (0.95-0.99) | 0.01 |
| Baseline proteinuria | 1.01 (0.96 – 1.06) | 0.64 |
| AKI at diagnosis | 1.10 (0.25 – 4.06) | 0.98 |
| Hypertension | 0.25 (0.05 – 1.33) | 0.11 |
| Diabetes mellitus | 1.76 (1.48 – 2.48) | 0.39 |

*Sensitivity multivariable Cox regression model including baseline comorbidities (hypertension and diabetes). P 0.02, the multivariate model is globally significant. Baseline kidney function remained independently associated with kidney survival, while treatment status, baseline proteinuria, AKI, hypertension and diabetes were not significantly associated with progression to ESKD.*

*Abbreviations: HR, hazard ratio; CI, confidence interval; eGFR, estimated glomerular filtration rate; AKI, acute kidney injury; ESKD, end-stage kidney disease.*

**Supplementary Table 4. Multivariable Cox regression analysis between treatment and kidney survival, including baseline histological variables**

| Variable | HR (CI 95%) | P |
| --- | --- | --- |
| Treatment | 0.47 (0.09 – 2.36) | 0.33 |
| Glomerular sclerosis (%) | 1.00 (0.95 – 1.06) | 0.92 |
| Interstitial fibrosis | 1.61 (0.64 – 4.03) | 0.31 |
| Vascular amyloid deposition | 0.34 (0.05 – 2.08) | 0.23 |

*Multivariable Cox regression model including treatment status and baseline histological variables. P 0.49, the multivariable model is not globally significant and none of the histological parameters evaluated were independently associated with kidney survival.*

**Supplementary Table 5. Kidney response in patients treated by decades of diagnosis**

|  | 1990-2000 | 2000-2010 | 2010-2024 | p |
| --- | --- | --- | --- | --- |
| N = 76 | 19 (25) | 20 (26) | 37 (49) | - |
| Patients treated | 8 (42) | 15 (75) | 35 (95) | 0.01 |
| Patients not treated | 11 (58) | 5 (25) | 2 (5) | 0.01 |
| Kidney remission | 3 (16) | 5 (25) | 22 (60) | 0.02 |
| Time to kidney remission, median (IQR) | 9 (3 – 9) | 9 (6 – 15) | 6 (3 – 18) | 0.85 |

*Data is presented as N (%) unless otherwise specified***.**

**Supplementary Table 6. Multivariable Cox regression analysis for predictors of kidney survival**

| Variable | HR (CI 95%) | P |
| --- | --- | --- |
| Kidney remission | 0.15 (0.03-0.78) | 0.02 |
| eGFR at diagnosis (mL/min/1.73m^2^) | 0.97 (0.96-0.98) | 0.01 |
| Hematologic remission | 0.45 (0.19-1.05) | 0.06 |
| Baseline proteinuria (g/day) | 1.12 (0.97-1.30) | 0.11 |
| Age at diagnosis (years) | 1.01 (0.98-1.04) | 0.40 |

*Kidney remission and higher eGFR at diagnosis were independently associated with improved kidney survival. HR are shown with their corresponding 95% CI and p-values. A HR < 1 indicates a lower risk of progression. Hematologic remission, baseline proteinuria, and age at diagnosis did not show a significant association with kidney survival in the adjusted model.*

*Abbreviations: HR, hazard ratio; CI, confidence interval; eGFR, estimated glomerular filtration rate.*

**Supplementary table 7. Lines of hematological treatment received according to time to kidney** **response stratified by the time to hematological response**

|  |  |  | Kidney response | | | p |
| --- | --- | --- | --- | --- | --- | --- |
|  |  | **N** | **Very early**  **≤ 6 months** | **Early**  **6-12 months** | **Late**  **> 12 months** |  |
| Hematologic response | **Very early**  **≤ 6 months**  Lines of treatment  1  2  3 | 21  13  3  5 | 6 (28%)  3  2  1 | 11 (53%)  9  1  1 | 4 (19%)  1  0  3 | 0.04 |
|  | **Early**  **6-12 months**  Lines of treatment  1  2  3 | 4  2  1  1 | 0  0  0  0 | 1 (25%)  1  0  0 | 3 (75%)  1  1  1 | 0.51 |
|  | **Late**  **>12 months**  Lines of treatment  1  2  3 | 5  2  2  1 | 1 (20%)  1  0  0 | 1 (20%)  0  1  0 | 3 (75%)  1  1  1 | 0.50 |
|  | **Total** | 30 | 7 (23%) | 13 (43%) | 10 (34%) |  |
